# Topics of Public Interest

**Published:** 1910-03

**Authors:** 


### TOPICS OF PUBLIC INTEREST

### The State Charities Aid Association Circular of February 21, 1910

### Buffalo Tests Law—Finds Registration of Tuberculosis an Excellent Protection

A REPORT from Dr. F. E. Fronczak, the acting Health Commissioner of Buffalo, received today by the State Charities Aid Association, shows some striking results of the work of the inspectors recently appointed in that city for the purpose of more adequately enforcing the tuberculosis law of 1908. The report covers a period of three months, from the appointment of the inspector, on October 1 to December 31, 1909.

It shows 347 individual houses inspected, 290 houses investigated after removals and 117 after deaths of persons having tuberculosis. There were made 159 disinfections and 153 fumigations to prevent the spread of the disease. But 540 living cases of tuberculosis were registered in 1907; while in 1908 after the law drafted by the State Charities Aid Association there were 740 living cases reported and in 1909 the number increased to 1883, more than doubling the reported living cases in two years. Of this 1,183, 452 were reported since the appointment of the inspectors 1st of October. In 1909 there were 523 deaths from tuberculosis as compared with 535 in 1908 which reductions may be due partly to better supervision of living cases. Already there are more than two living cases of tuberculosis reported for every death, whereas two years ago the deaths greatly exceeded the reported living cases.

These inspectors were appointed largely through the efforts of Acting Health Commissioner Fronczak, and Mr. John R. Shillady, Executive Secretary of the Buffalo Association for the Relief and Control of Tuberculosis. The law above mentioned under which they are operating declares tuberculosis to be an infectious and communicable disease. It provides for the free examination of sputum, and for the registration by the physician of all the living cases of tuberculosis. The records of such cases are protected from the public by a special provision of the law. Disinfection by the owner of the premises is compulsory.

The section which health authorities regard as most important is that which details the precautions to be taken by the physician and the health department on the premises of a tuberculosis patient, for the protection of other persons occupying the same premises. Physicians may either carry into effect such procedure and precaution, for which they will receive a fee of one dollar, or if unwilling or unable to take such steps, shall so certify to the health authorities and the duty thereupon devolves upon the health authorities who for the performance thereof shall receive a similar sum. It is required that the health authorities keep on hand the prophylactic supplies necessary to carry out the provisions of this section, and these supplies are furnished free to the patients needing them.

The test in Buffalo is of interest all over the State. There are now 57 cities and villages systematically organised to bring about measures for the cure and prevention of tuberculosis. Over 3,000 of the leading citizens of the state representing financial, religious, professional, social, and industrial groups are demanding adequate registration.

---

### Tuberculosis Sunday

---

**A National Tuberculosis Sunday—Churches Are Being Enlisted in Consumption Crusade.**

Announcement of a national tuberculosis Sunday to be held April 24, 1910, in 215,000 churches of the United States was made February 24, by the National Association for the Study and Prevention of Tuberculosis.

Following campaigns against consumption that have been carried on in the churches of hundreds of cities, and sermons on tuberculosis that have been preached before thousands of congregations during the past year, a movement has been started to establish a permanent tuberculosis Sunday, on which it is hoped that every one of the 33,000,000 churchgoers in the United States will hear the gospel of health. It is planned to enlist the active co-operation of anti-tuberculosis organisations, labor unions, fraternal organisations, and other bodies together with the churches in the movement. The aid of leading churchmen in many of the principal denominations has already been offered. All of the large interdenominational bodies, such as the Young Men's Christian Association, the Young Women's Christian Association, the King's Daughters and Sons, and the various young people's societies are also in sympathy with the anti-tuberculosis campaign.

It is planned that on April 24, tuberculosis sermons shall be preached in all the churches of the country. Literature will be distributed to members of the congregations, and in every way an effort will be made to teach that tuberculosis is a dangerous disease and that it can be prevented and cured.

Clergymen who desire to obtain additional information in regard to tuberculosis will be able to secure literature from state and local anti-tuberculosis associations and boards of health, as well as from the National Association.

---

ACCORDING to a recent special despatch to the *World* from Albany, consent has been given by the State Insurance Department for a novel departure in the life insurance business. The Metropolitan Life Insurance Company asked for approval of a plan to buy a large plot of ground in Westchester County for a hospital, where it could take care of its employees afflicted with tuberculosis.

The company's application showed that it has a working force of about 14,000, a number of whom have tuberculosis and others of whom are threatened with it.

At first Superintendent Hotchkiss doubted that he had the power to decide the purchase of such land by the company was "necessary for its convenient accommodation in the transaction of its business," as provided by the law. The Supreme Court was asked in a friendly suit to determine whether he had the discretion to approve of the purchase.

The Appellate Division recently reported that the superintendent had such discretion under the law. Accordingly he determined the question on its merits in favor of the company. It is expected the company eventually may extend the scope of the institution to include policyholders.

